# The influence of early-life animal exposure on the risk of childhood atopic dermatitis, asthma and allergic rhinoconjunctivitis: findings from the Danish National Birth Cohort

**DOI:** 10.1093/ije/dyad040

**Published:** 2023-04-05

**Authors:** Angela Pinot De Moira, Neil Pearce, Marie Pedersen, Anne-Marie Nybo Andersen

**Affiliations:** Section of Epidemiology, Department of Public Health, University of Copenhagen, Copenhagen, Denmark; National Heart and Lung Institute, Imperial College London, London, UK; Department of Medical Statistics, London School of Hygiene and Tropical Medicine, London, UK; Section of Epidemiology, Department of Public Health, University of Copenhagen, Copenhagen, Denmark; Section of Epidemiology, Department of Public Health, University of Copenhagen, Copenhagen, Denmark

**Keywords:** Lifecourse epidemiology, pets, animals, atopic dermatitis, asthma, allergic rhinoconjunctivitis, allergic disease, Danish National Birth Cohort, children

## Abstract

**Background:**

Early-life animal exposure has been associated with both protective and harmful effects on asthma and allergic disease. We aimed to explore factors that may modify associations of early-life animal exposure with asthma and allergic disease, so as to better understand these differences in findings.

**Methods:**

We used data from ≤84 478 children from the Danish National Birth Cohort recruited during pregnancy between 1996 and 2002, and linked registry data up to the child’s 13th birthday. Adjusted Cox models were used to examine associations of early-life cat, dog, rabbit, rodent, bird and livestock exposure with atopic dermatitis, asthma and allergic rhinoconjunctivitis overall, and by source of exposure (domestic or occupation), parental history of asthma or allergy, maternal education level and timing of exposure.

**Results:**

Overall, associations between animal exposure and the three outcomes of interest were weak. However, dog exposure was associated with marginally lower risk of atopic dermatitis and asthma [adjusted hazard ratio (aHR) = 0.81, 95% CI: 0.70–0.94 and 0.88, 95% CI: 0.82–0.94, respectively], whereas prenatal domestic bird exposure was associated with slightly increased risk of asthma (aHR = 1.18, 95% CI: 1.05–1.32). Source of exposure, parental history of asthma or allergy and timing of exposure modified associations. Early-life animal exposure did not appear to increase the risk of allergic rhinoconjunctivitis (aHR range = 0.88, 95% CI: 0.81–0.95 to 1.00, 95% CI: 0.91–1.10).

**Conclusions:**

The overall weak associations observed between animal exposure and atopic dermatitis, asthma and allergic rhinoconjunctivitis were modified by type of animal, source of exposure, parental history of asthma or allergy and timing of exposure, suggesting that these factors should be considered when assessing the risks associated with early-life animal exposure.

Key Messages

- We used data from a large prospective birth cohort to examine associations of early-life animal exposure with childhood atopic dermatitis, asthma and allergic rhinoconjunctivitis, including potential modifying factors.
- Overall, associations between animal exposure and the three outcomes of interest were weak.
- Early-life dog exposure was associated with marginally lower risk of atopic dermatitis and asthma, whilst early-life bird exposure was associated with marginally increased risk of asthma.
- There was no evidence of early-life animal exposure increasing the risk of allergic rhinoconjunctivitis.
- Source of exposure, parental history of asthma or allergy and timing of exposure were observed to modify associations, suggesting that these factors should be considered when assessing the risks associated with early-life animal exposure.

## Introduction

Atopic dermatitis (AD), asthma and allergic rhinoconjunctivitis (AR) are complex, multifactorial diseases that commonly co-exist as multiple morbidities^1^ and share similar, although not identical, risk factors.^2^ The prevalence of these diseases has increased in recent decades and continues to increase in many countries.^3–5^ These increases are thought to be in part driven by changes in lifestyle and environmental exposures resulting in reduced or delayed exposure to microorganisms.^6^

Studies have shown that exposure to animals can increase the diversity of the gut microbiome^7^^,^^8^ and thus it has been suggested that early-life animal exposure could support the development of the immune system^9^^,^^10^ and protect against immune dysregulatory diseases such as AD, asthma and AR. However, findings from studies investigating the influence of early-life animal exposure on later risk of asthma and allergic disease have been inconsistent.^11–13^ Several factors that have been found to modify associations of early-life animal exposure with these disease outcomes could explain observed inconsistencies. These include the development of allergic sensitization,^14^ the prevalence of animal keeping in a community^15^ and duration and timing of exposure.^16–18^

Although most studies have focused on the risks associated with cats and dogs or ‘furry pets’ as a single entity, it is also likely that associated risks will vary depending on the type of animal. For example, animals such as rabbits, rodents and birds are less popular as pets (and therefore less prevalent),^19^ have different associated allergens^20^ and differ with respect to pet-keeping practices and their lifespan. Further possible sources of variation include, but are not limited to, the extent of animal contact outside the home^21^ and the tendency for families with asthma or allergy to actively avoid pets, which could bias results.^22^

The aim of the current study was to comprehensively examine associations of early-life animal exposure with later risk of AD, asthma and AR, including potential modifying factors, in a large prospective birth cohort. We specifically examine: (i) overall associations between prenatal species-specific animal exposure and risk of AD, asthma and AR; (ii) whether associations vary by source of exposure; (iii) the possibility of reverse causation; and (iv) the relative influence of prenatal vs early-childhood exposure.

## Methods

### Study population

The Danish National Birth Cohort (DNBC) is a nationwide birth cohort study that was established between 1996 and 2002.^23^ Danish-speaking women who intended to carry their pregnancy to term were enrolled into the study by informed consent during their first antenatal visit with their general practitioner (GP). Approximately 100 000 expectant mothers were recruited into the study; ∼60% of all women were invited by their GP and 30% of eligible pregnancies. Mothers were invited to participate in two pre-natal telephone interviews, with further follow-ups conducted when children were 6 and 18 months, and 7, 11, 14 and 18 years.

**Figure 1:**
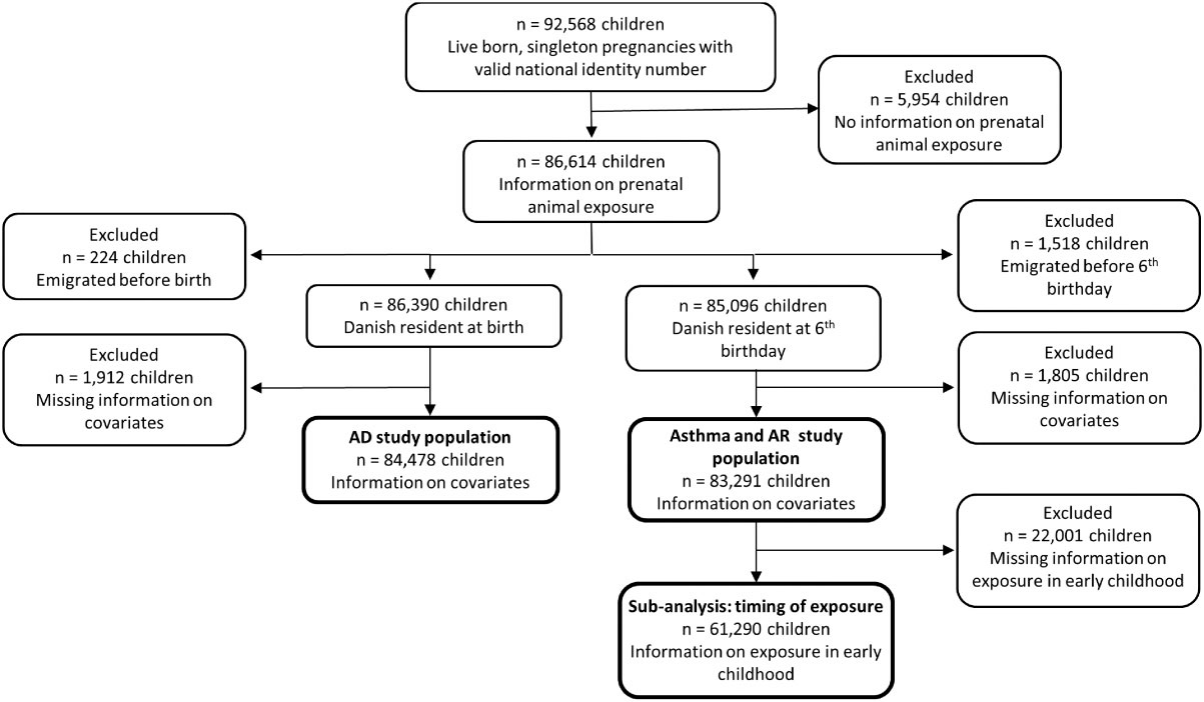
Flow chart illustrating participants included in each analysis. AD, atopic dermatitis; AR, allergic rhinoconjunctivitis

The current study includes all live-born singleton children participating in the DNBC with information on maternal animal exposure during pregnancy and relevant covariates (Figure 1). Participants included in analyses were comparable to those in the original study population (Supplementary Table S1, available as Supplementary data at *IJE* online).

### Outcomes: AD, asthma and AR

Children with AD, asthma and AR were identified using linked hospital episode (inpatient and ambulatory) and/or prescription data held in the Danish National Patient Register^24^ and the Danish National Prescription Registry,^25^ respectively. Children were identified as having AD if they had an AD-specific record in the Danish National Patient Register according to the International Classification of Diseases code, 10th revision (ICD-10 code L20), as previously validated in the Danish population.^26^ Asthma and AR were based on ICD-10 codes or at least two disease-specific prescriptions dispensed within a 12-month period and selected exclusion criteria, following algorithms validated in the Danish population (detailed in Supplementary Information Part 1, available as Supplementary data at *IJE* online).^27^^,^^28^

In sensitivity analyses, we used the following outcome definitions based on parental-reported symptoms and/or disease outcomes obtained from questionnaires: AD derived from a validated algorithm created for DNBC at 18 months;^29^ current asthma at 7 years according to the MeDALL (Mechanisms of the Development of Allergy) definition;^30^ AR determined by using the International Study of Asthma and Allergies in Childhood-based question: ‘Has [child’s name] ever suffered from sneezing/running or blocked nose, even though [child’s name] did not have a cold or influenza?’, asked at the 11-year follow-up. Further details are provided in Supplementary Table S2 (available as Supplementary data at *IJE* online).

### Exposures

Information on mothers’ animal exposure was obtained during the first prenatal telephone interview, at ∼16 weeks’ gestation, where mothers were asked about their domestic (pet), occupational and farm-related contact with animals, including type of animal (questions are provided in Supplementary Information Part 2, available as Supplementary data at *IJE* online).

These data were used to create six binary variables (yes/no) capturing combined domestic, occupational or farm-related prenatal exposure to dogs, cats, rabbits, rodents, birds and livestock. Six categorical variables were also created detailing source of exposure for each animal group [none/farm or domestic (pet)/occupation for livestock, and none/domestic (pet)/other (occupation or farm) for all other animal groups].

Information on the index child’s exposure to animals in the first 2 years of life was obtained during the 18-month telephone interview, in which mothers were asked to name any animals the child was in contact with (detailed in Supplementary Information Part 2, available as Supplementary data at *IJE* online). These data were combined with information on prenatal animal exposure to create categorical variables relating to the timing of cat, dog, rabbit, rodent and bird exposure (never/prenatal only/early-childhood only/both prenatal and early-childhood).

### Covariates

Potential confounders were identified based on the literature and the causal model represented in a directed acyclic graph (Supplementary Figure S1, available as Supplementary data at *IJE* online). Potential confounders included: (i) maternal asthma (yes/no); (ii) maternal inhalant allergy (yes/no); (iii) paternal asthma (yes/no); (iv) paternal allergy (yes/no); (v) maternal education (low/medium/high); (vi) quartiles of equivalized disposable household income the year prior to the child’s birth; (vii) maternal age at birth; (viii) number of children living in the home (0/1/≥2); (ix) household crowding (≤0.5/>0.5–1/>1 person per room); (x) smoking during pregnancy (yes/no); (xi) living in Copenhagen at birth (yes/no); (xii) sex.

Information on maternal and paternal asthma and allergy, the number of children living in the home, household crowding and maternal smoking were obtained from prenatal telephone interviews conducted at ∼16 and ∼31 weeks’ gestation. Only information on any allergy as opposed to specifically inhalant allergy was available for fathers. Maternal education was based on the mother’s highest completed educational level recorded in the Danish Population’s Education Register^31^ at the time of the child’s birth, categorized into three groups according to the International Standard Classification of Education (ISCED) 2011: low (ISCED 0–2), medium (ISCED 3–4) and high (ISCED 5–8).^32^ Equivalized disposable household income was obtained from the Income Statistics register^33^ and categorized into year-specific quartiles. Maternal age at birth was obtained from the Danish Medical Birth Registry.^34^ Finally, the Population Statistics Register was used to identify mothers living in the municipality of Copenhagen (code 101), the most urbanized region of Denmark.

### Statistical analyses

**Figure 2:**
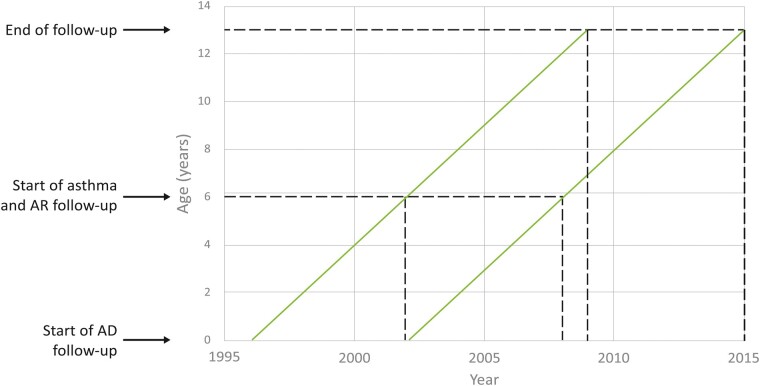
Lexis diagram displaying the start and end of follow-up for each analysis. AR, allergic rhinoconjunctivitis; AD, atopic dermatitis

Associations between animal exposures and AD, asthma and AR were examined separately using Cox proportional hazard models, using age as the underlying timescale. Due to differences in the age these conditions first occur or can be diagnosed, the start dates of follow-up varied according to the outcome (Figure 2). For AD, children were followed from birth until: (i) first occurrence of AD; (ii) migration; (iii) death; (iv) their 13th birthday. For asthma and AR, children were followed from their sixth birthday until: (i) first occurrence of asthma or AR; (ii) migration; (iii) death; (iv) their 13th birthday.

Date of disease debut was defined as the first date of either a specific ICD-10 code or of a dispensed prescription.

We performed analyses first with minimal adjustment for age and sex only, then with additional adjustment for the confounders detailed above and mutual adjustment for other animal exposures. We included age and sex in minimally adjusted models due to their wide association with most health outcomes, thus enabling more informative comparisons after adjusting for other covariates. We assessed collinearity between exposures and covariates by examining changes in mean squared errors;^35^ no strong collinearity was observed. To assess the possibility of reverse causation, we examined effect modification by parental history of asthma or allergy and maternal education. In order to determine whether associations differed between girls and boys, we also stratified analyses by sex. Finally, because AD, asthma and AR tend to be associated^2^^,^^36^ and share similar risk factors,^2^ we performed an interaction analysis to determine whether the presence of AD modified associations of animal exposure with asthma and AR.

The validity of the proportional hazards assumption was assessed by plotting Kaplan–Meier curves and log–log plots, and calculating scaled Schoenfeld residuals to test the independence between residuals and time. Where the proportional hazards assumption was violated, stratified Cox models were used to adjust for covariates with non-proportional hazards. Robust standard errors were calculated to allow for within-family dependency.

All analyses were conducted in Stata version 14.2.

### Sensitivity analyses

In sensitivity analyses, we explored the sensitivity of findings to the applied definition of AD, asthma and AR. For these analyses, questionnaire-derived and register-based outcomes were analysed using logistic regression, restricted to the same children (detailed in Supplementary Table S2, available as Supplementary data at *IJE* online).

## Results

Selected characteristics of singletons included in the DNBC with information on prenatal animal exposure are displayed in Table 1, overall and by animal exposure. Children with pre-natal animal exposure tended to have a lower household income, lower maternal education and lower prevalence of parental allergy than children without exposure. Exposed children were more likely to live in a household with at least two children and less likely to live in Copenhagen. Other characteristics varied depending on the type of animal exposure. For example, mothers of children exposed to cats, dogs, rodents or birds were more likely to smoke during pregnancy, whereas those of children exposed to rabbits, rodents, birds or livestock were more likely to be multiparous (Table 1).

**Table 1:** Characteristics of the study population, overall and by prenatal animal exposure

	All^a^	Cat	Dog	Rabbit	Rodent	Bird	Livestock
	*N* = 86 614	*N* = 20 199	*N* = 19 388	*N* = 3872	*N* = 2063	*N* = 6101	*N* = 5313
**Child**							
Atopic dermatitis [*n* (%)]	1258 (1.5)	255 (1.3)	233 (1.2)	59 (1.5)	33 (1.6)	76 (1.3)	54 (1.0)
Missing	223 (0.3)	44 (0.2)	44 (0.2)	13 (0.3)	11 (0.5)	24 (0.4)	7 (0.1)
Asthma [*n* (%)]	5796 (6.7)	1251 (6.2)	1169 (6.0)	275 (7.1)	145 (7.0)	442 (7.2)	317 (6.0)
Missing	1499 (1.7)	241 (1.2)	214 (1.1)	47 (1.2)	36 (1.8)	68 (1.1)	53 (1.0)
Allergic rhinoconjunctivitis [*n* (%)]	19 581 (22.6)	4371 (21.6)	4144 (21.4)	724 (18.7)	437 (21.2)	1263 (20.7)	1016 (19.1)
Missing	1396 (1.6)	235 (1.2)	200 (1.0)	46 (1.2)	33 (1.6)	66 (1.1)	52 (1.0)
Female [*n* (%)]	42 217 (48.7)	9833 (48.7)	9447 (48.7)	1818 (47.0)	976 (47.3)	2897 (47.5)	2600 (48.9)
**Parents’ characteristics**							
Maternal age at birth [mean (SD)]	30 (4.3)	30 (4.5)	30 (4.5)	31 (4.7)	30 (5.3)	30 (4.7)	30 (4.4)
Multiparous [*n* (%)]	45 681 (52.7)	10 716 (53.1)	10 585 (54.6)	3051 (78.8)	1422 (68.9)	3859 (63.3)	3409 (64.2)
Education [*n* (%)]							
Low	11 387 (13.2)	3326 (16.5)	3456 (17.8)	766 (19.8)	568 (27.5)	1227 (20.1)	787 (14.8)
Medium	41 172 (47.5)	10 328 (51.1)	10 550 (54.4)	1906 (49.2)	927 (44.9)	2916 (47.8)	2709 (51.0)
High	33 733 (39.0)	6493 (32.2)	5336 (27.5)	1192 (30.8)	561 (27.2)	1933 (31.7)	1806 (34.0)
Missing	322 (0.4)	52 (0.3)	46 (0.2)	8 (0.2)	7 (0.3)	25 (0.4)	11 (0.2)
Smoked in pregnancy [*n* (%)]	23 141 (26.7)	6263 (31.0)	5837 (30.1)	1112 (28.7)	770 (37.3)	1851 (30.3)	1207 (22.7)
Missing	505 (0.6)	94 (0.5)	100 (0.5)	32 (0.8)	11 (0.5)	33 (0.5)	23 (0.4)
Maternal history of asthma [*n* (%)]	7528 (8.7)	1611 (8.0)	1698 (8.8)	352 (9.1)	205 (9.9)	589 (9.7)	389 (7.3)
Missing	28 (0.03)	5 (0.02)	<5	<5	<5	<5	<5
Maternal history of allergy [*n* (%)]	26 972 (31.1)	5695 (28.2)	5597 (28.9)	1208 (31.2)	627 (30.4)	1939 (31.8)	1453 (27.4)
Missing	183 (0.2)	47 (0.2)	39 (0.2)	12 (0.3)	<5	15 (0.3)	9 (0.2)
Maternal history of inhalent allergy [*n* (%)]	14 500 (16.7)	2769 (13.7)	2901 (15.0)	635 (16.4)	306 (14.8)	993 (16.3)	711 (13.4)
Missing	266 (0.3)	68 (0.3)	57 (0.3)	15 (0.4)	<5	16 (0.3)	12 (0.2)
Maternal history of animal allergy [*n* (%)]	4349 (5.0)	565 (2.8)	724 (3.7)	166 (4.3)	100 (4.9)	296 (4.9)	173 (3.3)
	266 (0.3)	68 (0.3)	57 (0.3)	15 (0.4)	<5	16 (0.3)	12 (0.2)
Paternal history of asthma [*n* (%)]	7095 (8.2)	1385 (6.9)	1538 (7.9)	312 (8.1)	183 (8.9)	535 (8.8)	336 (6.3)
Missing	619 (0.7)	174 (0.9)	125 (0.6)	15 (0.4)	21 (1.0)	43 (0.7)	27 (0.5)
Paternal history of allergy [*n* (%)]	20 227 (23.4)	3705 (18.3)	3812 (19.7)	811 (21.0)	439 (21.3)	1258 (20.6)	878 (16.5)
Missing	8 (0.01)	<5	<5	0	0	0	<5
**Home characteristics**							
Household income [*n* (%)]							
1 ‘Quintile 1 (low)’	21 162 (24.4)	5525 (27.4)	5046 (26.0)	1239 (32.0)	858 (41.6)	1980 (32.5)	2084 (39.2)
4 ‘Quintile 4 (high)’	21 644 (25.0)	4015 (19.9)	3792 (19.6)	522 (13.5)	271 (13.1)	926 (15.2)	819 (15.4)
Missing	78 (0.1)	7 (0.03)	8 (0.04)	<5	<5	<5	<5
Number of children in HH [*n* (%)]							
≥2	14 039 (16.2)	3988 (19.7)	3875 (20.0)	1701 (43.9)	797 (38.6)	1794 (29.4)	1620 (30.5)
Missing	46 (0.1)	10 (0.05)	7 (0.04)	<5	<5	0	<5
Crowding (persons/room) [*n* (%)]							
≤0.5	27 411 (31.7)	7507 (37.2)	8066 (41.6)	879 (22.7)	419 (20.3)	1904 (31.2)	2516 (47.4)
>1	3911 (4.5)	828 (4.1)	723 (3.7)	258 (6.7)	180 (8.7)	369 (6.1)	229 (4.3)
Missing	142 (0.2)	42 (0.2)	33 (0.2)	6 (0.2)	<5	10 (0.2)	13 (0.2)
Copenhagen [*n* (%)]	8406 (9.7)	1139 (5.6)	377 (1.9)	145 (3.7)	109 (5.3)	261 (4.3)	12 (0.2)
Missing	77 (0.1)	7 (0.03)	8 (0.04)	<5	<5	<5	12 (0.1)
Source of exposure [*n* (%)]							
Domestic (pet)^b^		20 034 (99.2)	19 314 (99.6)	3771 (97.4)	1983 (96.1)	4059 (66.5)	5179 (97.5)
Other^c^		165 (0.8)	74 (0.4)	101 (2.6)	80 (3.9)	2042 (33.5)	134 (2.5)
Timing of exposure [*n* (%)]							
Prenatal only		3181 (15.8)	1622 (8.4)	1975 (51.0)	850 (41.2)	2873 (47.1)	
Prenatal and early-childhood		11 669 (57.8)	12 527 (64.6)	886 (22.9)	608 (29.5)	1594 (26.1)	
Missing		5349 (26.5)	5239 (27.0)	1011 (26.1)	605 (29.3)	1634 (26.8)	

Values are *n* (percent) or mean (standard deviation). SD, standard deviation; HH, household.

a All live-born, singleton children with information on prenatal animal exposure.

b Domestic or farm for livestock-related exposures.

c Occupation (livestock), and occupation and farm exposures (all other animal groups).

### Associations of prenatal animal exposure with AD, asthma and AR

Overall, effect estimates were relatively small for all prenatal animal exposures (Table 2). Prenatal exposure to dogs was associated with a marginally lower risk of AD and asthma [adjusted hazard ratio (aHR) = 0.81, 95% CI: 0.70–0.94 and 0.88, 95% CI: 0.82–0.94, respectively], whereas prenatal bird exposure was associated with a marginally higher risk of asthma (aHR = 1.12, 95% CI: 1.00–1.24). Associations of prenatal animal exposure with AR indicated either no change or a very slightly protective effect (Table 2). Findings were similar for girls and boys (Supplementary Table S3, available as Supplementary data at *IJE* online). Interestingly, any protective effect of animal exposure on asthma or AR was negated if the child developed AD (Supplementary Table S4, available as Supplementary data at *IJE* online). In some instances risks were elevated, notably for associations of early-life rabbit or livestock exposure with asthma (aHR = 1.71, 95% CI: 1.10–2.66 and 1.69, 95% CI: 1.03–2.76, respectively) (Supplementary Table S4, available as Supplementary data at *IJE* online).

**Table 2:** Associations of prenatal animal exposure with atopic dermatitis, asthma and allergic rhinoconjunctivitis

		Atopic dermatitis			Asthma			Allergic rhinoconjunctivitis		
		Cases	Minimally adjusted^a^ HR (95% CI)	Adjusted^b^ HR (95% CI)	Cases	Minimally adjusted^a^ HR (95% CI)	Adjusted^b^ HR (95% CI)	Cases	Minimally adjusted^a^ HR (95% CI)	Adjusted^b^ HR (95% CI)
Cat	No	974	–	–	4435	–	–	14 902	–	–
	Yes	251	0.84 (0.73,0.97)	0.93 (0.80,1.07)	1219	0.89 (0.84,0.95)	0.94 (0.88,1.01)	4272	0.92 (0.89,0.96)	0.97 (0.93,1.00)
Dog	No	1000	–	–	4509	–	–	15 102	–	–
	Yes	225	0.77 (0.67,0.89)	0.81 (0.70,0.94)	1145	0.86 (0.81,0.92)	0.88 (0.82,0.94)	4072	0.91 (0.88,0.94)	0.93 (0.90,0.97)
Rabbits	No	1168	–	–	5389	–	–	18 465	–	–
	Yes	57	1.04 (0.79,1.36)	1.16 (0.88,1.52)	265	1.03 (0.91,1.17)	1.10 (0.96,1.24)	709	0.79 (0.73,0.85)	0.88 (0.81,0.95)
Rodents	No	1193	–	–	5513	–	–	18 747	–	–
	Yes	32	1.10 (0.77,1.56)	1.17 (0.82,1.67)	141	1.05 (0.89,1.24)	1.06 (0.90,1.26)	427	0.92 (0.84,1.02)	1.00 (0.91,1.10)
Birds	No	1152	–	–	5220	–	–	17 938	–	–
	Yes	73	0.83 (0.65,1.05)	0.90 (0.70,1.14)	434	1.09 (0.98,1.20)	1.12 (1.00,1.24)	1236	0.88 (0.83,0.94)	0.95 (0.90,1.01)
Livestock	No	1174	–	–	5342	–	–	18 169	–	–
	Yes	51	0.65 (0.49,0.87)	0.79 (0.59,1.06)	312	0.88 (0.78,0.98)	1.00 (0.88,1.13)	1005	0.82 (0.77,0.87)	0.93 (0.87,0.99)

HR, hazard ratio.

a Adjusted for age (underlying time scale) and sex.

b Additionally adjusted for maternal asthma, maternal inhalant allergy, paternal asthma, paternal allergy, maternal education, equivalized disposable household income, maternal age at birth, number of children living in the home, household crowding, smoking during pregnancy and living in Copenhagen, plus mutually adjusted for other animal exposures.

In sensitivity analyses, results were largely consistent for reported and register-based outcomes (Supplementary Table S2, available as Supplementary data at *IJE* online).

### Source of animal exposure and risk of AD, asthma and AR

Associations were mainly unchanged when analyses were restricted to domestic (pet) exposures (Table 3), which is reflective of pets being the predominant source of exposure (Table 1). The only exception was for associations of prenatal bird exposure and asthma, which strengthened. Occupational and farm-related bird exposures were not associated with AD or asthma, but were associated with slightly lower risk of AR (Table 3).

**Table 3:** Influence of source of exposure on associations of animal exposure with atopic dermatitis, asthma and allergic rhinoconjunctivitis

		Atopic dermatitis		Asthma		Allergic rhinoconjunctivitis	
		Minimally adjusted^a^ HR (95% CI)	Adjusted^b^ HR (95% CI)	Minimally adjusted^a^ HR (95% CI)	Adjusted^b^ HR (95% CI)	Minimally adjusted^a^ HR (95% CI)	Adjusted^b^ HR (95% CI)
Cat	No exposure	–	–	–	–	–	–
	Domestic	0.84 (0.73,0.97)	0.92 (0.80,1.06)	0.89 (0.83,0.95)	0.95 (0.89,1.01)	0.92 (0.89,0.96)	0.97 (0.94,1.00)
	Other^c^	NA	NA	1.23 (0.71,2.12)	1.36 (0.76,2.43)	0.90 (0.65,1.26)	1.20 (0.84,1.70)
Dog	No exposure	–	–	–	–	–	–
	Domestic	0.77 (0.67,0.89)	0.81 (0.69,0.94)	0.86 (0.81,0.92)	0.88 (0.82,0.95)	0.91 (0.88,0.94)	0.93 (0.90,0.97)
	Other^c^	NA	NA	NA	NA	0.40 (0.19,0.84)	0.42 (0.19,0.92)
Rabbit	No exposure	–	–	–	–	–	–
	Domestic	1.04 (0.79,1.37)	1.16 (0.88,1.53)	1.03 (0.91,1.17)	1.09 (0.96,1.24)	0.79 (0.73,0.85)	0.88 (0.81,0.95)
	Other^c^	NA	NA	1.05 (0.50,2.23)	1.28 (0.58,2.82)	0.69 (0.42,1.14)	0.90 (0.54,1.47)
Rodent	No exposure	–	–	–	–	–	–
	Domestic	1.07 (0.75,1.54)	1.13 (0.78,1.63)	1.05 (0.89,1.24)	1.06 (0.89,1.26)	0.93 (0.84,1.02)	1.00 (0.90,1.10)
	Other^c^	NA	NA	0.94 (0.39,2.26)	0.96 (0.39,2.38)	0.84 (0.51,1.41)	1.04 (0.61,1.76)
Birds	No exposure	–	–	–	–	–	–
	Domestic	0.86 (0.64,1.14)	0.85 (0.64,1.13)	1.19 (1.06,1.34)	1.18 (1.05,1.32)	0.97 (0.91,1.04)	1.00 (0.93,1.07)
	Other^c^	0.78 (0.51,1.17)	0.99 (0.64,1.54)	0.88 (0.73,1.05)	0.95 (0.78,1.16)	0.72 (0.65,0.80)	0.83 (0.74,0.93)
Livestock	No exposure	–	–	–	–	–	–
	Domestic and farm	0.66 (0.50,0.87)	0.78 (0.58,1.06)	0.86 (0.77,0.97)	1.01 (0.88,1.15)	0.81 (0.76,0.87)	0.95 (0.88,1.02)
	Other^d^	NA	NA	1.43 (0.81,2.52)	1.52 (0.86,2.69)	0.95 (0.64,1.40)	1.02 (0.69,1.50)

HR, hazard ratio; NA, effect estimates not available due to risk of disclosure.

a Adjusted for age (underlying time scale) and sex.

b Additionally adjusted for maternal asthma, maternal inhalant allergy, paternal asthma, paternal allergy, maternal education, equivalized disposable household income, maternal age at birth, number of children living in the home, household crowding, smoking during pregnancy and living in Copenhagen, plus mutually adjusted for other animal exposures.

c Farm- and occupation-related exposure.

d Occupation-related exposure.

### Exploring reverse causation

Since evidence suggests that families with asthma or allergies may avoid pets^22^ and children with a family history of asthma or allergy are also at increased risk of asthma, avoidance of pets may result in an apparent protective effect of pets in the whole population. To explore this potential for reverse causation, we examined whether associations were modified by parental history of asthma or allergy. Except for associations of rodent exposure with AD and AR, and of livestock exposure with AD, there was a weak overall tendency of lower hazard ratios (HR) among children with parental history of asthma or allergy compared with children without (Table 4). This tendency was slightly stronger for associations of cat and dog exposure with asthma, of rabbit exposure with AD and asthma, and of bird exposure with AD (Table 4). In the case of rabbits and birds, exposure was associated with slightly higher rates of AD and asthma among children without parental history of asthma or allergy but there was no association among children with parental history (Table 4). A slightly lower proportion of poultry-related exposures were reported for children with parental history of asthma or allergy compared with those without but otherwise the types of birds children were prenatally exposed to were largely similar for these two groups (Supplementary Table S5, available as Supplementary data at *IJE* online).

**Table 4:** Associations of prenatal animal exposure with atopic dermatitis, asthma and allergic rhinoconjunctivitis by reported family history of asthma or allergy

	Atopic dermatitis, adjusted^a^ HR (95% CI)			Asthma, adjusted^a^ HR (95% CI)			Allergic rhinoconjunctivitis, adjusted^a^ HR (95% CI)		
	No family history	Family history	*P*_interaction_	No family history	Family history	*P*_interaction_	No family history	Family history	*P*_interaction_
Cat	0.97 (0.80,1.19)	0.85 (0.69,1.04)	0.33	1.01 (0.92,1.10)	0.86 (0.78,0.94)	0.02	0.99 (0.95,1.04)	0.92 (0.87,0.97)	0.03
Dog	0.84 (0.68,1.04)	0.78 (0.63,0.96)	0.59	0.94 (0.85,1.04)	0.83 (0.76,0.91)	0.06	0.95 (0.90,1.00)	0.90 (0.86,0.95)	0.17
Rabbit	1.60 (1.13,2.25)	0.78 (0.50,1.23)	0.01	1.23 (1.03,1.47)	0.98 (0.82,1.17)	0.07	0.90 (0.81,0.99)	0.85 (0.76,0.96)	0.53
Rodent	1.01 (0.58,1.74)	1.30 (0.82,2.07)	0.48	1.10 (0.86,1.41)	1.03 (0.82,1.30)	0.70	0.98 (0.86,1.12)	1.02 (0.88,1.17)	0.75
Bird	1.14 (0.83,1.57)	0.70 (0.49,1.01)	0.05	1.18 (1.02,1.37)	1.09 (0.95,1.25)	0.43	0.98 (0.91,1.07)	0.92 (0.84,1.00)	0.25
Livestock	0.73 (0.49,1.09)	0.85 (0.56,1.28)	0.60	1.01 (0.86,1.19)	0.96 (0.80,1.15)	0.65	0.94 (0.86,1.03)	0.90 (0.81,1.00)	0.51

HR, hazard ratio.

a Adjusted for age (underlying timescale), sex, maternal education, equivalized disposable household income, maternal age at birth, number of children living in the home, household crowding, smoking during pregnancy and living in Copenhagen, plus mutually adjusted for other animal exposures.

We explored the potential for reverse causation further by examining whether maternal education modified associations. Knowledge acquired through education may affect receptiveness to medical advice,^37^ meaning that mothers with a higher level of education may be more likely to avoid pets if they or their children are at increased risk of asthma or allergies. However, we observed no evidence that maternal education modified associations (*P*_interaction_ > 0.10) (Supplementary Table S6, available as Supplementary data at *IJE* online).

### Timing of animal exposure and risk of asthma and AR

In a subset of children with information on both prenatal and early-childhood animal exposure (*n* = 61 290, Figure 1), we examined whether the life stage in which exposure occurs influences the risk of asthma and AR (Table 5). For cats and dogs, the majority of exposures occurred both prenatally and in early childhood (Table 1). Exposure to cats or dogs only in prenatal life was not associated with asthma or AR, but exposure during early childhood only or continuously in early life was associated with slightly reduced risk.

**Table 5:** Influence of timing of exposure on associations of animal exposure with asthma and allergic rhinoconjunctivitis

		Asthma			Allergic rhinoconjunctivitis		
		Cases	Minimally adjusted^a^ HR (95% CI)	Adjusted^b^ HR (95% CI)	Cases	Minimally adjusted^a^ HR (95% CI)	Adjusted^b^ HR (95% CI)
Cat	Never	3112	–	–	10 540	–	–
	Pregnancy only	207	0.96 (0.83,1.10)	0.99 (0.85,1.14)	683	0.93 (0.86,1.01)	0.99 (0.91,1.07)
	Early-childhood only	124	0.80 (0.67,0.96)	0.85 (0.71,1.02)	424	0.79 (0.72,0.88)	0.85 (0.77,0.94)
	Pregnancy and early-childhood	701	0.88 (0.81,0.95)	0.94 (0.86,1.02)	2501	0.92 (0.88,0.96)	0.95 (0.91,1.00)
Dog	Never	3198	–	–	10 861	–	–
	Pregnancy only	114	1.03 (0.86,1.25)	1.06 (0.88,1.29)	377	1.01 (0.92,1.12)	1.06 (0.95,1.17)
	Early-childhood only	107	0.94 (0.78,1.14)	0.96 (0.79,1.17)	299	0.75 (0.67,0.84)	0.78 (0.69,0.87)
	Pregnancy and early-childhood	725	0.84 (0.78,0.91)	0.87 (0.80,0.94)	2611	0.88 (0.85,0.92)	0.89 (0.85,0.93)
Rabbit	Never	3883	–	–	13 324	–	–
	Pregnancy only	132	1.00 (0.84,1.19)	1.07 (0.90,1.28)	364	0.79 (0.71,0.87)	0.89 (0.80,0.99)
	Early-childhood only	69	0.72 (0.57,0.91)	0.76 (0.60,0.97)	301	0.91 (0.81,1.02)	1.01 (0.90,1.14)
	Pregnancy and early-childhood	60	1.00 (0.78,1.30)	1.07 (0.83,1.39)	159	0.76 (0.65,0.89)	0.84 (0.71,0.98)
Rodent	Never	3987	–	–	13 655	–	–
	Pregnancy only	51	0.91 (0.69,1.20)	0.96 (0.72,1.26)	182	0.96 (0.82,1.11)	1.07 (0.92,1.24)
	Early-childhood only	55	0.86 (0.66,1.12)	0.91 (0.70,1.19)	172	0.76 (0.65,0.88)	0.84 (0.72,0.98)
	Pregnancy and early-childhood	51	1.28 (0.97,1.69)	1.30 (0.98,1.73)	139	1.01 (0.85,1.19)	1.07 (0.91,1.27)
Bird	Never	3761	–	–	12 996	–	–
	Pregnancy only	202	1.07 (0.93,1.23)	1.12 (0.97,1.30)	564	0.84 (0.77,0.91)	0.92 (0.84,1.00)
	Early-childhood only	70	0.90 (0.71,1.14)	0.92 (0.72,1.16)	251	0.93 (0.82,1.05)	0.98 (0.86,1.11)
	Pregnancy and early-childhood	111	1.07 (0.88,1.29)	1.07 (0.89,1.30)	337	0.93 (0.83,1.04)	0.97 (0.87,1.08)

HR, hazard ratio.

a Adjusted for age (underlying timescale) and sex.

b Additionally adjusted for maternal asthma, maternal inhalant allergy, paternal asthma, paternal allergy, maternal education, equivalized disposable household income, maternal age at birth, number of children living in the home, household crowding, smoking during pregnancy and living in Copenhagen, plus mutually adjusted for other animal exposures.

For rabbits, rodents and birds, the majority of exposures occurred prenatally only (Table 1). This possibly reflects the wording of the 18-month interview whereby mothers were specifically asked about pets the child was in contact with (Supplementary Information Part 2, available as Supplementary data at *IJE* online) and also the large proportion of farm and occupationally related prenatal bird exposures (Table 1). The influence of timing of exposure on the risk of asthma or AR was less clear for these animals but there was some indication of an association between prenatal bird exposure and asthma (Table 5).

## Discussion

In this study of ≤84 537 children from the DNBC, we used detailed information on early-life animal exposure, including type of animal, source and timing of exposure, and linked population-based register data to examine how early-life animal exposure influences the risk of AD, asthma and AR. Overall, effect estimates for animal exposure were relatively small. We did, however, observe evidence that early-life exposure to dogs may offer some slight protection against AD and asthma, but that prenatal exposure to birds may slightly increase the risk of asthma. We also observed evidence that source of exposure, parental history of asthma or allergy and timing of exposure may modify associations. Associations with AR were weak but did not indicate any harmful effect of early-life animal exposure.

The strengths of this study include its large size, prospective design and detailed information on prenatal animal exposures, encompassing domestic, occupational and farm-related exposures, as well as exposure to animals other than cats and dogs, which have been less studied. We based our outcome measures on linked hospital episode and/or disease-specific prescription data, which allowed complete follow-up, thereby minimizing selection bias due to non-participation. Using registry-based outcomes likely increased the specificity of our outcome measures,^38^ particularly for AD, which is often confused with other eczemas such as contact eczema by caregivers. Linkage with registry data also allowed us to obtain accurate measures of socio-economic background, which, due to their sensitive nature, are prone to information bias when self-reported. We were also able to adjust for a number of other confounders using interview or linked registry data.

However, our study also has several limitations. First, our register-based AR outcome will lack sensitivity to identify milder cases of the disease that can be treated using over-the-counter medication. This may explain the small effect estimates observed for AR. Similarly, due to the lack of specificity of medication used to treat AD, we based our definition of AD solely on ICD-10 codes and will have missed milder cases of AD. Nonetheless, effect estimates for AD were similar to those obtained using caregiver-reported symptoms in sensitivity analyses. We also did not consider different disease phenotypes in our analyses, which may have different environmental associations.^39^^,^^40^ In addition, we lacked data on pet-keeping practices and allergic sensitization status, both of which may modify associations of animal exposure on asthma and allergic disease.^14^ Finally, since our study was only conducted in the Danish population, within a relatively homogenous study population, our results may not be generalizable to other populations.

The literature regarding the influence of early-life animal exposure on later risk of asthma and allergic disease is conflicting.^11^^,^^12^^,^^41–44^ Studies generally point towards early-life cat and dog exposure having no or a slightly protective effect on AD, asthma and AR,^41^^,^^44–46^ as did our findings, but also that the prevalence of cat or dog keeping in a community^15^^,^^45^^,^^47^ and the development of allergic sensitization^14^ may modify associations. Specifically, evidence suggests that lower prevalence of pet keeping or the development of pet-specific allergic sensitization increases the risks associated with pet keeping.^14^^,^^15^^,^^45^^,^^47^

The literature regarding the effects of early-life exposure to rabbits, rodents and birds on the risk of asthma and allergic disease is sparser. A meta-analysis of European birth cohort data found no association between rodent or bird exposure and school-age asthma or AR,^44^ whereas a UK birth cohort study observed associations between rabbit and rodent, but not bird, ownership and increased odds of non-atopic asthma^43^ and persistent wheeze.^13^ Other studies have observed evidence that childhood bird ownership may increase the odds of adult wheeze^47^ but others that early-life poultry exposure may have a protective effect against allergic sensitization^48^ and AD.^49^ We did not observe evidence of a protective effect of birds against AD but did observe evidence of a positive association between early-life bird exposure and asthma. This might relate to avian antigens found in feathers, bloom, serum and droppings, which have been associated with respiratory symptoms, including hypersensitivity pneumonitis^50^ and asthma.^51^ Associations of rabbit and rodent exposure pointed towards either no or a positive association with AD and asthma, and no or an inverse association with AR. This contrasts slightly with our findings for cat and dog exposure, possibly reflecting the lower prevalence of ownership for these pets.^15^ Associations of animal exposure also differed among children who developed AD compared with those who did not; a possible explanation for this could be the disrupted skin barrier and sustained T-helper 2 inflammation associated with AD increasing the risk of developing allergic sensitization to animal-associated allergens.^52^

The tendency we observed for animal exposure to be associated with lower rates of AD, asthma and AR among children with parental history of asthma or allergy compared with children without could be consistent with reverse causation, namely the avoidance of pets by families with pet allergies,^22^ or differences in the types of pets owned and pet-keeping practices between these two groups. The latter of these could affect allergen and microbial exposures, particularly if pets are kept outside.^53^ Indeed, occupational bird exposures, which occur outside the home, were not associated with asthma, but domestic bird exposures were positively associated with asthma. Unfortunately, we did not have data on pet avoidance due to asthma or allergies or pet-keeping practices to verify this. Findings from the International Study of Asthma and Allergies in Childhood, though, provide evidence against reverse causation for associations of cat exposure with wheeze.^54^ We also did not observe evidence of maternal education modifying associations. An alternative explanation for our findings is gene–environment interactions. For instance, exposure to cats or dogs in early life has been associated with reduced risk of asthma^55^ and allergic sensitization^56^ among children with polymorphisms in the 17q12–21 region and filaggrin gene, respectively. These polymorphisms are otherwise respectively associated with early-onset asthma and impaired skin barrier, eczema and allergic sensitization.

In conclusion, whereas overall our findings do not indicate a strong role for early-life animal exposure in the development of AD and asthma, they do point towards early-life dog exposure offering some slight protection against AD and asthma, and bird exposure increasing the risk of asthma. Source of exposure, parental history of asthma or allergy and timing of exposure may also modify risks. Our results were more consistent for AR and did not suggest an increased risk with early-life animal exposure.

## Ethics approval

The DNBC is approved by the Danish Data Protection Agency and the Committee on Health Research Ethics. The DNBC participants were enrolled by informed consent. This study received approval from University of Copenhagen Faculty of Health and Medical Sciences under case number 514–0538/20–3000.

## Supplementary Material

dyad040_Supplementary_Data

## Data Availability

The code used in this study is available on Github (https://github.com/angelapinotdemoira/DNBC_animals_asthma_allergy.git). Due to restrictions in Danish law, the confidential healthcare data used in this study can only be accessed through Statistics Denmark, the state organization holding the rights to the data. Danish scientific organizations can be authorized to work with data within Statistics Denmark and can provide access to individual scientists inside and outside of Denmark. Data are available via the Research Service Department at Statistics Denmark (www.dst.dk/da/TilSalg/Forskningsservice) for researchers who meet the criteria for access to confidential data. The authors of this study had no special access privileges that others would not have.
