# Supplementary material for: The influence of early-life animal exposure on the risk of childhood atopic dermatitis, asthma and allergic rhinoconjunctivitis: findings from the Danish National Birth Cohort
Source: Int J Epidemiol. 2023 Apr 5;52(4):1231–42. doi: 10.1093/ije/dyad040 (PMC10396419; doi:10.1093/ije/dyad040)
Supplement: dyad040_Supplementary_Data [file dyad040_supplementary_data.docx]

**
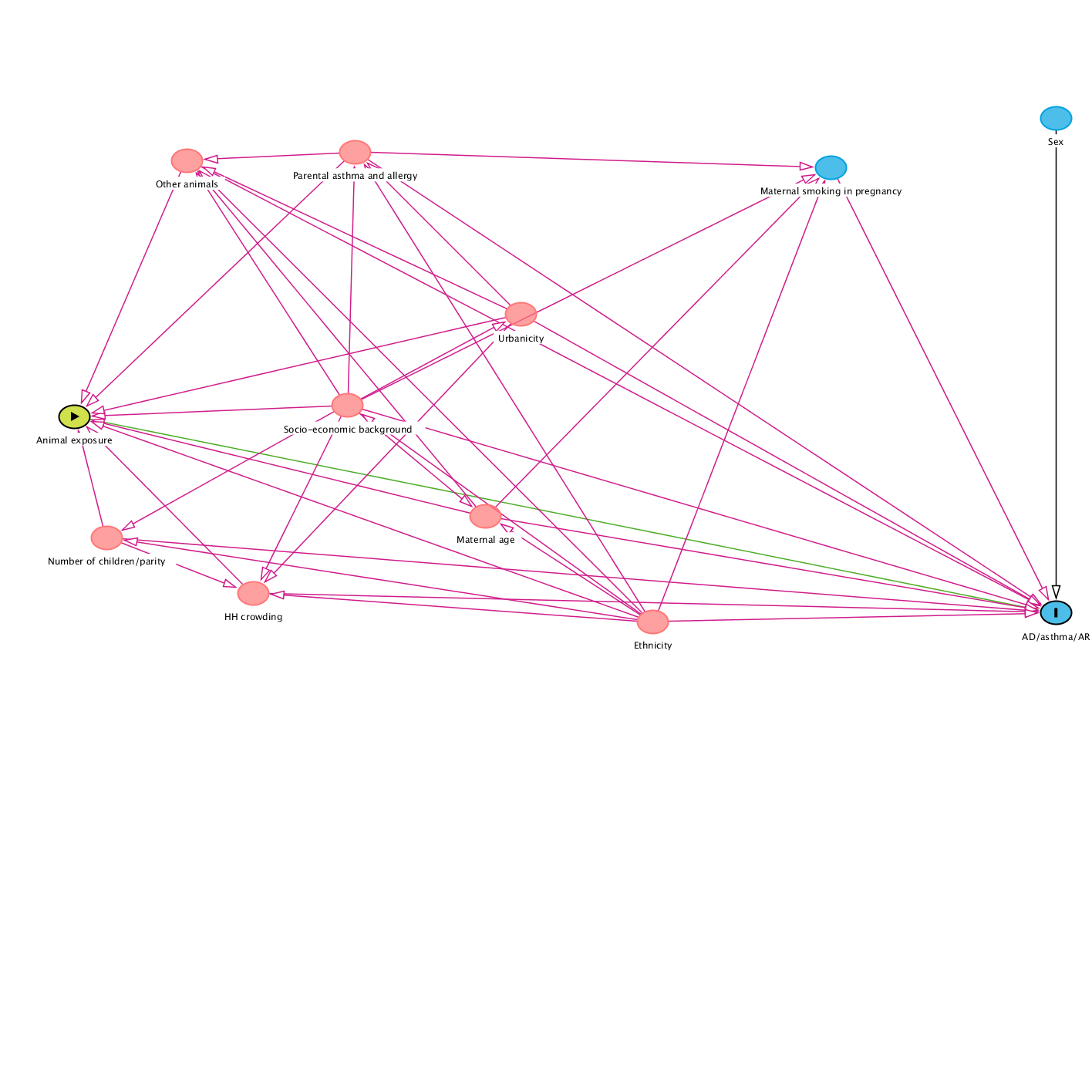
Supplementary Material**

**Supplementary Figure S1.** Simplified directed acyclic graph depicting the minimum set of variables required to control for confounding between prenatal animal exposure and atopic dermatitis (AD)/asthma/allergic rhinoconjunctivitis (AR). Here, the green circle with the triangle is the main exposure (animal exposure in pregnancy) and the blue circle with the “I” is the outcome (AD/asthma/AR). Potential confounders are depicted by red circles. Variables that are not expected to be associated with pet exposure but are related to the outcome asthma are depicted by blue circles. Arrows denote the direction of causal association.

Maternal smoking in pregnancy and sex are unlikely to be confounders but were included in models due to their close relationship with the outcome, to increase the precision of effect estimates.

**Supplementary Table S1** Characteristics of the original DNBC study population, atopic dermatitis (AD) study population, asthma and allergic rhinoconjunctivitis (AR) study population, and timing of exposure study population (depicted in Figure 1).

|  | **All liveborn singletons** | **AD study population** | **Asthma and AR study population** | **Timing of exposure study population** |
| --- | --- | --- | --- | --- |
|  | **N=92,568** | **N=84,478** | **N=83,291** | **N=61,290** |
| **Child** |  |  |  |  |
| Atopic dermatitis, n (%) | 1,334 (1.4) | 1,225 (1.5) | 1,219 (1.5) | 928 (1.5) |
| Asthma, n (%) | 6,173 (6.8) | 5,648 (6.8) | 5,654 (6.8) | 4,144 (6.8) |
| Allergic rhinoconjunctivitis, n (%) | 20,916 (23.0) | 19,140 (23.0) | 19,174 (23.0) | 14,148 (23.1) |
| Female, n (%) | 45,133 (48.8) | 41,150 (48.7) | 40,564 (48.7) | 29,992 (48.9) |
|  |  |  |  |  |
| **Parents’ characteristics** | |  |  |  |
| Maternal age at birth, mean (SD) | 30 (4.3) | 30 (4.3) | 30 (4.3) | 30 (4.2) |
| Multiparous, n (%) | 48,659 (52.6) | 44,829 (53.1) | 44,313 (53.2) | 33,245 (54.2) |
| Education, n (%) |  |  |  |  |
| Low | 12,398 (13.5) | 11,079 (13.1) | 10,965 (13.2) | 7,094 (11.6) |
| Medium | 43,791 (47.5) | 40,398 (47.8) | 39,913 (47.9) | 29,648 (48.4) |
| High | 35,983 (39.0) | 33,001 (39.1) | 32,413 (38.9) | 24,548 (40.1) |
| Smoked in pregnancy, n (%) | 23,960 (26.6) | 22,579 (26.7) | 22,306 (26.8) | 15,699 (25.6) |
| Maternal history of asthma, n (%) | 7,535 (8.7) | 7,329 (8.7) | 7,220 (8.7) | 5,109 (8.3) |
| Maternal history of allergy, n (%) | 26,993 (31.2) | 26,264 (31.1) | 25,918 (31.1) | 19,036 (31.1) |
| Maternal history of inhalent allergy, n (%) | 14,514 (16.8) | 14,198 (16.8) | 14,021 (16.8) | 10,309 (16.8) |
| Maternal history of animal allergy, n (%) | 4,352 (5.0) | 4,252 (5.0) | 4,188 (5.0) | 3,058 (5.0) |
| Paternal history of asthma, n (%) | 7,099 (8.2) | 6,961 (8.2) | 6,861 (8.2) | 4,986 (8.1) |
| Paternal history of allergy, n (%) | 20,242 (23.4) | 19,792 (23.4) | 19,477 (23.4) | 14,366 (23.4) |
|  |  |  |  |  |
| **Home characteristics** |  |  |  |  |
| Household income |  |  |  |  |
| 1 "Quintile 1 (low)" | 22,994 (24.9) | 20,463 (24.2) | 20,101 (24.1) | 13,704 (22.4) |
| 2 "Quintile 2" | 23,270 (25.2) | 21,462 (25.4) | 21,256 (25.5) | 15,880 (25.9) |
| 3 "Quintile 3" | 23,136 (25.0) | 21,369 (25.3) | 21,166 (25.4) | 16,089 (26.3) |
| 4 "Quintile 4 (high)" | 23,065 (24.9) | 21,184 (25.1) | 20,768 (24.9) | 15,617 (25.5) |
| Number of children in HH |  |  |  |  |
| 0 | 39,926 (46.1) | 38,743 (45.9) | 38,110 (45.8) | 27,441 (44.8) |
| 1 | 32,661 (37.7) | 32,007 (37.9) | 31,599 (37.9) | 23,540 (38.4) |
| >=2 | 14,049 (16.2) | 13,728 (16.3) | 13,582 (16.3) | 10,309 (16.8) |
| Crowding (persons/room) |  |  |  |  |
| <=0.5 | 27,434 (31.7) | 26,773 (31.7) | 26,376 (31.7) | 19,678 (32.1) |
| >0.5-1 | 55,194 (63.8) | 53,924 (63.8) | 53,224 (63.9) | 39,115 (63.8) |
| >1 | 3,912 (4.5) | 3,781 (4.5) | 3,691 (4.4) | 2,497 (4.1) |
| Copenhagen, n (%) | 9,091 (9.8) | 8,115 (9.6) | 7,881 (9.5) | 5,378 (8.8) |
| Prenatal cat exposure, n (%) | 20,213 (23.3) | 19,720 (23.3) | 19,531 (23.4) | 14,467 (23.6) |
| Prenatal dog exposure, n (%) | 19,400 (22.4) | 18,977 (22.5) | 18,810 (22.6) | 13,824 (22.6) |
| Prenatal rabbit exposure, n (%) | 3,872 (4.5) | 3,777 (4.5) | 3,750 (4.5) | 2,784 (4.5) |
| Prenatal rodent exposure, n (%) | 2,063 (2.4) | 2,004 (2.4) | 1,981 (2.4) | 1,413 (2.3) |
| Prenatal bird exposure, n (%) | 6,106 (7.0) | 5,947 (7.0) | 5,909 (7.1) | 4,346 (7.1) |
| Prenatal livestock exposure, n (%) | 5,317 (6.1) | 5,214 (6.2) | 5,172 (6.2) | 3,871 (6.3) |

Values are n (percent) or mean (standard deviation)

Abbreviations: *AD* atopic dermatitis; *AR* allergic rhinoconjunctivitis AR; *SD* standard deviations; *HH* household

**Supplementary Table S2.** Results of sensitivity analyses examining the robustness of results to the definition of atopic dermatitis, asthma or allergic rhinoconjunctivitis applied.

|  | Atopic dermatitis^a^ | | Asthma^d^ | | Allergic rhinoconjunctivitis^f^ | |
| --- | --- | --- | --- | --- | --- | --- |
|  | Questionnaire-based^b^ OR^c^ (95%CI) | Registry-based OR^c^ (95% CI) | Questionnaire-based^e^ OR^c^ (95%CI) | Registry-based OR^c^ (95% CI) | Questionnaire-based^g^ OR^c^ (95%CI) | Registry-based OR^c^ (95% CI) |
| Cat | 0.94 [0.88,1.00] | 0.90 [0.75,1.08] | 0.94 [0.86,1.04] | 0.95 [0.89,1.02] | 0.95 [0.89,1.01] | 0.97 [0.91,1.03] |
| Dog | 0.78 [0.73,0.84] | 0.79 [0.65,0.95] | 0.95 [0.86,1.05] | 1.00 [0.93,1.08] | 0.83 [0.77,0.89] | 0.88 [0.83,0.94] |
| Rabbit | 1.20 [1.06,1.37] | 1.31 [0.93,1.84] | 1.11 [0.92,1.33] | 1.01 [0.88,1.16] | 0.98 [0.86,1.13] | 0.94 [0.82,1.07] |
| Rodent | 1.08 [0.91,1.29] | 0.94 [0.57,1.57] | 1.33 [1.06,1.66] | 1.05 [0.88,1.26] | 0.98 [0.81,1.18] | 1.08 [0.91,1.28] |
| Bird | 1.06 [0.95,1.18] | 0.87 [0.64,1.19] | 1.04 [0.89,1.21] | 1.06 [0.95,1.18] | 1.04 [0.94,1.16] | 0.92 [0.83,1.02] |
| Livestock | 0.92 [0.81,1.05] | 0.85 [0.60,1.23] | 1.03 [0.86,1.23] | 0.95 [0.83,1.09] | 0.83 [0.73,0.95] | 0.99 [0.88,1.11] |

Abbreviations: *OR* odds ratio; *CI* confidence interval

^a^ Analyses were restricted to children with questionnaire-derived measures of atopic dermatitis and who had not died or emigrated before the 18-month telephone interview (n=53,140).

^b^ Derived from a validated algorithm created for DNBC at 18 months which considers the presence, location, duration, recurrence, and treatment of itchy rash.^34^

^c^ Adjusted for sex, maternal asthma, maternal inhalant allergy, paternal asthma, paternal allergy, maternal education, equivalised disposable household income, maternal age at birth, number of children living in the home, household crowding, smoking during pregnancy and living in Copenhagen, plus mutually adjusted for other animal exposures.

^d^ Analyses were restricted to children with questionnaire-derived measures of asthma and who had not died or emigrated before 7-year follow-up (n=51,151).

^e^ According to the MeDALL (Mechanisms of the Development of Allergy) definition,^35^ whereby a child was classified as having current asthma when at least 2 of the following 3 criteria were reported: (1) doctor diagnosis of asthma ever, (2) use of asthma medication in the past 12 months, and (3) wheezing in the past 12 months according to the International Study of Asthma and Allergy in Childhood parental core questionnaire.^64^

^f^ Analyses were restricted to children with questionnaire-derived measures of allergic rhinoconjunctivitis and who had not died or emigrated before 11-year follow-up (n=42,021)

^g^ Based on the International Study of Asthma and Allergy in Childhood question: “Has [child name] ever suffered from sneezing/ running or blocked nose, even though [child name] did not have a cold or an influenza?”, asked at the 11-year FU.

**Supplementary Table S3.** Associations of prenatal animal exposure with atopic dermatitis, asthma and rhinoconjunctivitis stratified by sex^a^

|  | Atopic Dermatitis,  adjusted^b^ HR (95% CI) | |  | Asthma,  adjusted^b^ HR (95% CI) | |  | Allergic rhinoconjunctivitis,  adjusted^b^ HR (95% CI) | |
| --- | --- | --- | --- | --- | --- | --- | --- | --- |
|  | Male | Female |  | Male | Female |  | Male | Female |
| Cat | 0.97 [0.80,1.18] | 0.87 [0.71,1.08] |  | 0.96 [0.88,1.04] | 0.92 [0.83,1.03] |  | 0.99 [0.94,1.04] | 0.94 [0.89,0.99] |
| Dog | 0.88 [0.71,1.07] | 0.75 [0.60,0.94] |  | 0.88 [0.81,0.96] | 0.88 [0.79,0.98] |  | 0.95 [0.90,0.99] | 0.91 [0.86,0.96] |
| Rabbit | 1.32 [0.93,1.87] | 0.94 [0.60,1.47] |  | 1.09 [0.93,1.28] | 1.10 [0.90,1.36] |  | 0.85 [0.76,0.94] | 0.92 [0.82,1.03] |
| Rodent | 1.26 [0.80,1.98] | 1.02 [0.58,1.82] |  | 1.14 [0.93,1.40] | 0.93 [0.69,1.25] |  | 1.04 [0.91,1.18] | 0.96 [0.83,1.11] |
| Bird | 0.90 [0.65,1.24] | 0.88 [0.61,1.27] |  | 1.17 [1.03,1.33] | 1.03 [0.86,1.22] |  | 0.94 [0.87,1.02] | 0.96 [0.88,1.05] |
| Livestock | 0.85 [0.58,1.25] | 0.71 [0.45,1.12] |  | 0.87 [0.74,1.03] | 1.21 [1.00,1.47] |  | 0.91 [0.83,1.00] | 0.95 [0.86,1.05] |

Abbreviations: *HR* hazard ratio; *CI* confidence interval

^a^ Interaction was not tested due to violation of the proportional hazards assumption.

^b^ Adjusted for age (underlying time scale), maternal asthma, maternal inhalant allergy, paternal asthma, paternal allergy, maternal education, equivalised disposable household income, maternal age at birth, number of children living in the home, household crowding, smoking during pregnancy and living in Copenhagen, plus mutually adjusted for other animal exposures.

**Supplementary Table S4.** Influence of atopic dermatitis on associations of animal exposure with asthma and allergic rhinoconjunctivitis

|  | Asthma,  adjusted^a^ HR (95% CI) | | | Allergic rhinoconjunctivitis,  adjusted^a^ HR (95% CI) | | |
| --- | --- | --- | --- | --- | --- | --- |
|  | No AD | AD | P_interaction_ | No AD | AD | P_interaction_ |
| Cat | 0.89 [0.83,0.95] | 1.14 [0.87,1.51] | 0.08 | 0.94 [0.91,0.97] | 1.09 [0.88,1.35] | 0.18 |
| Dog | 0.86 [0.80,0.92] | 1.16 [0.87,1.54] | 0.04 | 0.92 [0.89,0.95] | 1.10 [0.88,1.39] | 0.13 |
| Rabbit | 1.06 [0.93,1.21] | 1.71 [1.10,2.66] | 0.04 | 0.86 [0.80,0.93] | 1.39 [0.95,2.03] | 0.02 |
| Rodent | 1.06 [0.89,1.27] | 0.95 [0.49,1.85] | 0.75 | 0.99 [0.90,1.10] | 1.00 [0.57,1.74] | 0.99 |
| Bird | 1.13 [1.02,1.26] | 1.47 [0.97,2.23] | 0.23 | 0.96 [0.90,1.02] | 0.97 [0.65,1.43] | 0.98 |
| Livestock | 0.94 [0.82,1.07] | 1.69 [1.03,2.76] | 0.02 | 0.91 [0.85,0.98] | 1.02 [0.63,1.65] | 0.65 |

Abbreviations: *HR* hazard ratio; *CI* confidence interval

^a^ Adjusted for age (underlying time scale), sex, maternal asthma, maternal inhalant allergy, paternal asthma, paternal allergy, maternal education, equivalised disposable household income, maternal age at birth, number of children living in the home, household crowding, smoking during pregnancy and living in Copenhagen, plus mutually adjusted for other animal exposures.

**Supplementary Table S5.** Details of rodents and birds included in exposure groups “rodent” and “bird”, overall and by parental history of asthma or allergy

|  | **All** | **No parental history** | **Parental History** |
| --- | --- | --- | --- |
| **Rodents, n (%)** |  |  |  |
| None | 84,544 (97.6) | 49,875 (97.5) | 34,669 (97.7) |
| Guinea pig | 1,132 (1.3) | 707 (1.4) | 425 (1.2) |
| Hamster | 478 (0.6) | 281 (0.5) | 197 (0.6) |
| Mouse or rat | 99 (0.1) | 60 (0.1) | 39 (0.1) |
| Chinchilla | 194 (0.2) | 113 (0.2) | 81 (0.2) |
| Other^a^ | 84 (0.1) | 45 (0.1) | 39 (0.1) |
| Undefined | 83 (0.1) | 47 (0.1) | 36 (0.1) |
|  |  |  |  |
| **Birds, n (%)** |  |  |  |
| None | 80,508 (93.0) | 47,455 (92.8) | 33,053 (93.1) |
| Undefined | 3,400 (3.9) | 1,949 (3.8) | 1,451 (4.1) |
| Indoor pet bird | 58 (0.1) | 33 (0.1) | 25 (0.1) |
| Poultry | 2,648 (3.1) | 1,691 (3.3) | 957 (2.7) |

Values are n (percent)

^a^ Gerbil, degu, jerboa, squirrel, chipmunk, unspecified.

**Table S6.** Associations of prenatal animal exposure with atopic dermatitis, asthma and allergic rhinoconjunctivitis stratified by maternal education level

|  | Atopic dermatitis, adjusted^a^ HR (95% CI) | | | |  | Asthma, adjusted^a^ HR (95% CI) | | | |  | Allergic rhinoconjunctivitis, adjusted^a^ HR (95% CI) | | | |
| --- | --- | --- | --- | --- | --- | --- | --- | --- | --- | --- | --- | --- | --- | --- |
|  | Low | Medium | High | P_interaction_ |  | Low | Medium | High | P_interaction_ |  | Low | Medium | High | P_interaction_ |
| Cat | 0.80 [0.55,1.15] | 0.93 [0.77,1.14] | 0.98 [0.76,1.26] | 0.56 |  | 0.90 [0.76,1.06] | 0.95 [0.86,1.04] | 0.97 [0.87,1.09] | 0.85 |  | 1.01 [0.93,1.11] | 0.95 [0.91,1.00] | 0.97 [0.91,1.04] | 0.40 |
| Dog | 0.73 [0.51,1.06] | 0.85 [0.69,1.04] | 0.79 [0.60,1.05] | 0.59 |  | 0.85 [0.72,1.01] | 0.87 [0.79,0.96] | 0.90 [0.80,1.03] | 0.94 |  | 0.97 [0.89,1.06] | 0.93 [0.89,0.98] | 0.90 [0.84,0.96] | 0.21 |
| Rabbit | 1.48 [0.85,2.58] | 1.18 [0.80,1.73] | 0.95 [0.55,1.65] | 0.56 |  | 1.22 [0.93,1.59] | 1.03 [0.85,1.24] | 1.12 [0.89,1.41] | 0.54 |  | 0.91 [0.77,1.08] | 0.81 [0.73,0.91] | 0.98 [0.85,1.12] | 0.17 |
| Rodent | 0.81 [0.38,1.76] | 1.20 [0.71,2.01] | 1.43 [0.76,2.70] | 0.61 |  | 0.97 [0.70,1.35] | 1.16 [0.91,1.48] | 1.00 [0.71,1.39] | 0.67 |  | 0.98 [0.82,1.18] | 1.00 [0.87,1.15] | 1.02 [0.84,1.24] | 0.99 |
| Birds | 1.15 [0.70,1.89] | 0.92 [0.65,1.30] | 0.72 [0.44,1.17] | 0.52 |  | 1.12 [0.89,1.40] | 1.22 [1.06,1.42] | 0.96 [0.78,1.17] | 0.11 |  | 0.92 [0.80,1.05] | 0.92 [0.84,1.00] | 1.02 [0.92,1.13] | 0.48 |
| Livestock | NA | 0.83 [0.56,1.24] | 0.97 [0.59,1.58] | 0.10 |  | 0.83 [0.59,1.15] | 1.04 [0.87,1.24] | 1.00 [0.80,1.25] | 0.53 |  | 0.94 [0.79,1.11] | 0.96 [0.88,1.06] | 0.87 [0.76,0.99] | 0.28 |

Abbreviations: *HR* hazard ratio; *CI* confidence interval; *NA* effect estimates not available due to risk of disclosure

^a^ Adjusted for age (underlying time scale), sex, maternal asthma, maternal inhalant allergy, paternal asthma, paternal allergy, equivalised disposable household income, maternal age at birth, number of children living in the home, household crowding, smoking during pregnancy and living in Copenhagen, plus mutually adjusted for other animal exposures.

**Supplementary Information Part 1A: algorithm to define children with asthma**

Children need to fulfil either criterion 1 or 2 below to be classified as having asthma:

**CRITERIA 1 (based on ICD-10 codes recorded in the Danish National Patient Register):**

≥1 records of:

J45.0”allergic asthma”

J45.1”non-allergic asthma”

J45.8 “asthma, different types”

J45.9 “asthma, unspecified”

J46.0 “status asthmaticus”

J46.9 “status asthmaticus, unspecified”

**CRITERIA 2 (based on ATC in the Danish National Prescription Registry):**

≥ 2 filled prescription of the following within a 12 month timeframe:

R03BA01 – R03BA08 “inhaled glucocorticoids”

R03DC01 – R03DC04 “leukotriene-receptor antagonists”

R03DC03 “montelukast (if no diagnosis of J30 allergic rhinitis)”

R03DB04 ”theophylline og adrenergics”

R03DA54 ”theophylline, combinations excl. psycholeptics”

R03BB01 ”anticholinergica, Ipratropium bromide”

R03DX05 ”omalizumab”

**Supplementary Information Part 1B: algorithm to define children with allergic rhinoconjunctivitis**

Children need to fulfil either criterion 1, 2, 3 or 4 below to be classified as having allergic rhinoconjunctivitis:

**CRITERIA 1 (based on ICD-10 codes recorded in the Danish National Patient Register):**

≥1 record of:

J30 “hay fever and allergic rhinitis”

J30.0 ”vasomotor rhinitis”

J30.1 “allergic rhinitis due to pollen”

J30.2 “other seasonal allergic rhinitis”

J30.3 “other allergic rhinitis”

J30.4 “allergic rhinitis, unspecified”

J31.0 “chronic rhinitis”

**CRITERIA 2 (based on ATC and ICD-10):**

≥ 2 filled prescriptions of the following recorded in the Danish National Prescription Registry:

R01AD01 – R01AD60 “inhaled corticosteroids for rhinitis”

And none of the following codes in the Danish National Patient Register (exclusions criteria):

J33 “nasal polyps”

J330 ”polyps in nasal cavity”

J331 “polyp related sinus degeneration”

J331A “woakes' ethmoiditis”

J338” nasal polyps, other”

J338A “polypus sinus sphenoidalis”

J339 “nasal polyps, unspecified”

J010- J019 ”acute sinusitis”

J320 –J329 ”chronic sinusitis”

**CRITERIA 3 (based on ATC and ICD-10)**

≥2 filled prescriptions of:

R06A “antihistamines for systemic use”

And none of the following codes in the Danish National Patient Register:

L29 “pruritus” or

DL50 “allergic urticaria”

**CRITERIA 4 (based on ATC in the Danish National Prescription Registry)**

≥ 1 filled prescriptions of:

V01A “specific immune therapy, allergen substract therapy” or/and

S01GX “medication for allergic conjunctivitis”

**Supplementary Information Part 2: DNBC questions relating to animal exposure**

***Prenatal telephone interview***

A201 Do you live in a farm with farm animals? (1 – yes; 2 – no; 3 - do not know; 4- do not want to answer; 9 – undefined; 10 – irrelevant)

A202 What animals?

A202_1 Horse *(1 – yes; 2 – no; 9 – undefined; 10 – irrelevant)*

A202_2 Cow *(1 – yes; 2 – no; 9 – undefined; 10 – irrelevant)*

A202_3 Pig *(1 – yes; 2 – no; 9 – undefined; 10 – irrelevant)*

A202_4 Poultry *(1 – yes; 2 – no; 9 – undefined; 10 – irrelevant)*

A202_5 Deer *(1 – yes; 2 – no; 9 – undefined; 10 – irrelevant)*

A202_6 Sheep *(1 – yes; 2 – no; 9 – undefined; 10 – irrelevant)*

A202_7 Other *(1 – yes; 2 – no; 9 – undefined; 10 – irrelevant)*

A202_8 Do not know *(1 – yes; 2 – no; 9 – undefined; 10 – irrelevant)*

A202_9 Do not want to answer *(1 – yes; 2 – no; 9 – undefined; 10 – irrelevant)*

A202A. Other, text

A203 Do you have any pets?

A203_1 fish *(1 – yes; 2 – no; 9 – undefined; 10 – irrelevant)*

A203_2 bird *(1 – yes; 2 – no; 9 – undefined; 10 – irrelevant)*

A203_3 pig *(1 – yes; 2 – no; 9 – undefined; 10 – irrelevant)*

A203_4 hamster *(1 – yes; 2 – no; 9 – undefined; 10 – irrelevant)*

A203_5 dog *(1 – yes; 2 – no; 9 – undefined; 10 – irrelevant)*

A203_6 rabbit *(1 – yes; 2 – no; 9 – undefined; 10 – irrelevant)*

A203_7 cat *(1 – yes; 2 – no; 9 – undefined; 10 – irrelevant)*

A203_8 reptiles *(1 – yes; 2 – no; 9 – undefined; 10 – irrelevant)*

A203_9 guinea pig *(1 – yes; 2 – no; 9 – undefined; 10 – irrelevant)*

A203_10 mouse *(1 – yes; 2 – no; 9 – undefined; 10 – irrelevant)*

A203_11 turtle *(1 – yes; 2 – no; 9 – undefined; 10 – irrelevant)*

A203_12 other *(1 – yes; 2 – no; 9 – undefined; 10 – irrelevant)*

A203_13 no *(1 – yes; 2 – no; 9 – undefined; 10 – irrelevant)*

A203_14 do not know *(1 – yes; 2 – no; 9 – undefined; 10 – irrelevant)*

A203_15 do not want to answer *(1 – yes; 2 – no; 9 – undefined; 10 – irrelevant)*

A203A Other pet, text

*AM093. Have you worked with animal farming during the pregnancy, ie with animals? (*1 – yes; 2 – no; 3 - do not know; 4- do not want to answer; 9 – undefined; 10 – irrelevant)

AM094. How much of the working time do you work with animals? (1 - almost all the time; 2 - 3/4 time; 3 - 1/2 time; 4 - 1/4 time; 5 - less often; 6 – never; 7 - don't know; 8 - don't want to answer; 9 – undefined; 10 – irrelevant)

*AM095. What animals do you work with?*

*AM095.1 Meat cattle (*1 – yes; 2 – no; 3 - do not know; 4- do not want to answer; 9 – undefined; 10 – irrelevant)

*AM095.2 Dairy cattle (*1 – yes; 2 – no; 3 - do not know; 4- do not want to answer; 9 – undefined; 10 – irrelevant)

*AM095.3 Pigs (*1 – yes; 2 – no; 3 - do not know; 4- do not want to answer; 9 – undefined; 10 – irrelevant)

*AM095.4 Chickens (*1 – yes; 2 – no; 3 - do not know; 4- do not want to answer; 9 – undefined; 10 – irrelevant)

*AM095.5 Turkey (*1 – yes; 2 – no; 3 - do not know; 4- do not want to answer; 9 – undefined; 10 – irrelevant)

*AM095.6 Sheep, lamb (*1 – yes; 2 – no; 3 - do not know; 4- do not want to answer; 9 – undefined; 10 – irrelevant)

*AM095.7 Horses (*1 – yes; 2 – no; 3 - do not know; 4- do not want to answer; 9 – undefined; 10 – irrelevant)

*AM095.8 Rodents (*1 – yes; 2 – no; 3 - do not know; 4- do not want to answer; 9 – undefined; 10 – irrelevant)

*AM095.9 Others (*1 – yes; 2 – no; 3 - do not know; 4- do not want to answer; 9 – undefined; 10 – irrelevant)

*AM095A If other, what? (1 – animals;* 2 - do not know; 3- do not want to answer; 4 – undefined; 5 – irrelevant*)*

*AM095B Type of animal, text*

*AM099 Have you worked at a slaughterhouse? (*1 – yes; 2 – no; 3 - do not know; 4- do not want to answer; 9 – undefined; 10 – irrelevant)

*AM100 Are you directly involved in handling animals at the slaughterhouse? (*1 – yes; 2 – no; 3 - do not know; 4 - do not want to answer; 9 – undefined; 10 – irrelevant)

*AM101 What animals do you work with?*

*AM101_1 cattle (1 – yes; 2 – no; 9 – undefined; 10 – irrelevant)*

*AM101_2 pigs (1 – yes; 2 – no; 9 – undefined; 10 – irrelevant)*

*AM101_3 poultry (1 – yes; 2 – no; 9 – undefined; 10 – irrelevant)*

*AM101_4 horses (1 – yes; 2 – no; 9 – undefined; 10 – irrelevant)*

*AM101_5 rodents (1 – yes; 2 – no; 9 – undefined; 10 – irrelevant)*

*AM101_6 others (1 – yes; 2 – no; 9 – undefined; 10 – irrelevant)*

*AM101_7 do not know (1 – yes; 2 – no; 9 – undefined; 10 – irrelevant)*

*AM101_8 do not want to answer (1 – yes; 2 – no; 9 – undefined; 10 – irrelevant)*

*AM101A Other, text*

*AM102 Have you worked with live animals? (*1 – yes; 2 – no; 3 - do not know; 4 - do not want to answer; 9 – undefined; 10 – irrelevant)

*AM105 What animals do you work with?*

*AM105_1 cattle (1 – yes; 2 – no; 9 – undefined; 10 – irrelevant)*

*AM105_2 pigs (1 – yes; 2 – no; 9 – undefined; 10 – irrelevant)*

*AM105_3 poultry (1 – yes; 2 – no; 9 – undefined; 10 – irrelevant)*

*AM105_4 horses (1 – yes; 2 – no; 9 – undefined; 10 – irrelevant)*

*AM105_5 rodents (1 – yes; 2 – no; 9 – undefined; 10 – irrelevant)*

*AM105_6 others (1 – yes; 2 – no; 9 – undefined; 10 – irrelevant)*

*AM105_7 do not know (1 – yes; 2 – no; 9 – undefined; 10 – irrelevant)*

*AM105_8 do not want to answer (1 – yes; 2 – no; 9 – undefined; 10 – irrelevant)*

*AM105A Other, text*

***18-month telephone interview***

D033.1-7 What animals or pets have you had since his/her birth?

(1 - No animals; 2 - Name of animal/pet: Library list (-> D033A); 3 - Yes, but animal not in contact with child: Only animals kept outside; 4 - No or answers/only animals that are not in contact with child; 5 - Do not know; 6 - Do not want to answer; 9 – Undefined; 10 – Irrelevant)

D033A.1-7 Types of animals and pets, text
